# Compartmentation of Redox Metabolism in Malaria Parasites

**DOI:** 10.1371/journal.ppat.1001242

**Published:** 2010-12-23

**Authors:** Sebastian Kehr, Nicole Sturm, Stefan Rahlfs, Jude M. Przyborski, Katja Becker

**Affiliations:** 1 Interdisciplinary Research Centre, Justus Liebig University, Giessen, Germany; 2 Department of Parasitology, Faculty of Biology, Philipps University Marburg, Marburg, Germany; La Trobe University, Australia

## Abstract

Malaria, caused by the apicomplexan parasite *Plasmodium*, still represents a major threat to human health and welfare and leads to about one million human deaths annually. *Plasmodium* is a rapidly multiplying unicellular organism undergoing a complex developmental cycle in man and mosquito – a life style that requires rapid adaptation to various environments. In order to deal with high fluxes of reactive oxygen species and maintain redox regulatory processes and pathogenicity, *Plasmodium* depends upon an adequate redox balance. By systematically studying the subcellular localization of the major antioxidant and redox regulatory proteins, we obtained the first complete map of redox compartmentation in *Plasmodium falciparum*. We demonstrate the targeting of two plasmodial peroxiredoxins and a putative glyoxalase system to the apicoplast, a non-photosynthetic plastid. We furthermore obtained a complete picture of the compartmentation of thioredoxin- and glutaredoxin-like proteins. Notably, for the two major antioxidant redox-enzymes – glutathione reductase and thioredoxin reductase – *Plasmodium* makes use of alternative-translation-initiation (ATI) to achieve differential targeting. Dual localization of proteins effected by ATI is likely to occur also in other *Apicomplexa* and might open new avenues for therapeutic intervention.

## Author Summary

The unicellular parasite *Plasmodium falciparum* is the causative agent of tropical malaria, which represents a global health burden. In order to survive in its human host and the malaria vector *Anopheles*, malaria parasites depend on adequate antioxidant defense and efficient redox regulation. Furthermore, as shown by glucose-6 phosphate dehydrogenase deficiency, a genetic variation protecting from malaria, redox equilibrium plays a vital role in parasite pathogenicity. Using a green fluorescent protein reporter gene, we systematically studied the subcellular compartmentation of redox networks in *Plasmodium falciparum*. Based on our results and data from literature, we provide the first thorough map of redox compartmentation. Most interestingly, for the two major antioxidant redox-enzymes – glutathione reductase (GR) and thioredoxin reductase (TrxR) – *Plasmodium falciparum* makes use of alternative translation initiation to translate protein isoforms with differing subcellular localization from one gene. Dual localization of proteins due to alternative translation initiation might occur frequently in *Apicomplexa* and identification of further genes that have evolved alternative translation initiation is likely to offer new therapeutic strategies against this devastating disease.

## Introduction

Malaria threatens more than 40% of the world's population. Current estimations point to 200–300 million clinical episodes and about 1 million human deaths each year [1]. The unicellular parasite *Plasmodium falciparum* causes the most dangerous form of this tropical disease including the development of cerebral malaria. Malaria parasites are continuously exposed to high fluxes of toxic reactive oxygen species (ROS) [2]. This is due to their life style in different intra- and extracellular environments, the high metabolic rate of the rapidly multiplying parasite, the intraparasitic haemoglobin digestion, and the ROS produced by the host's immune system [3]. In recent years *Plasmodium* has been shown to possess two major NADPH-dependent redox systems with a broad range of antioxidant defence mechanisms. This includes a complete glutathione system [2] comprising glutathione reductase [4] (GR), glutathione, glutaredoxin, and different glutaredoxin-like proteins [5], [6], glutathione S-transferase [7], and a functional glutathione dependent glyoxalase system [8]. Additionally, a complete thioredoxin system comprising thioredoxin reductase (TrxR), different thioredoxins and thioredoxin-like proteins, and thioredoxin-dependent peroxidases (TPx) has been characterised [2], [9], [10], [11]. Furthermore, two functional superoxide dismutases [12], [13], as well as two dihydrolipoamide dehydrogenase-like proteins [14], are present in the parasite. It has been demonstrated that malaria parasites are vulnerable to disruption of this redox equilibrium during their erythrocytic life stages [15]. This vulnerability is impressively underscored by the malaria-protective effects of glucose-6-phosphate dehydrogenase deficiency, one of the most frequent human gene defects worldwide, which leads to a lack of reducing equivalents provided by NADPH [16]. Two major antioxidant enzymes, catalase and a genuine glutathione peroxidase, do not occur in the parasite [2]. Taken together, this constellation offers great potential for the development of chemotherapeutic agents that act by perturbing the redox equilibrium of *Plasmodium* [3], [17], [18].

Many of the redox-active enzymes described above are predicted to contain protein targeting sequences. However, their localization has not yet been experimentally demonstrated. Although prediction algorithms have significantly improved over the years, experimental verification of protein localization is still essential. Predictions can often not be verified with regard to their significance and reliability, and multiple targeting of proteins has only rarely been considered by prediction methods [19]. In this study we systematically analyzed the subcellular compartmentation of the major redox-related proteins in *Plasmodium falciparum* (Access. No. provided in Table S1). Our data, together with published information, result in a comprehensive redox-map of the parasite.

## Results

### Mapping the subcellular localization of *P. falciparum* peroxiredoxins

**Figure 1:**
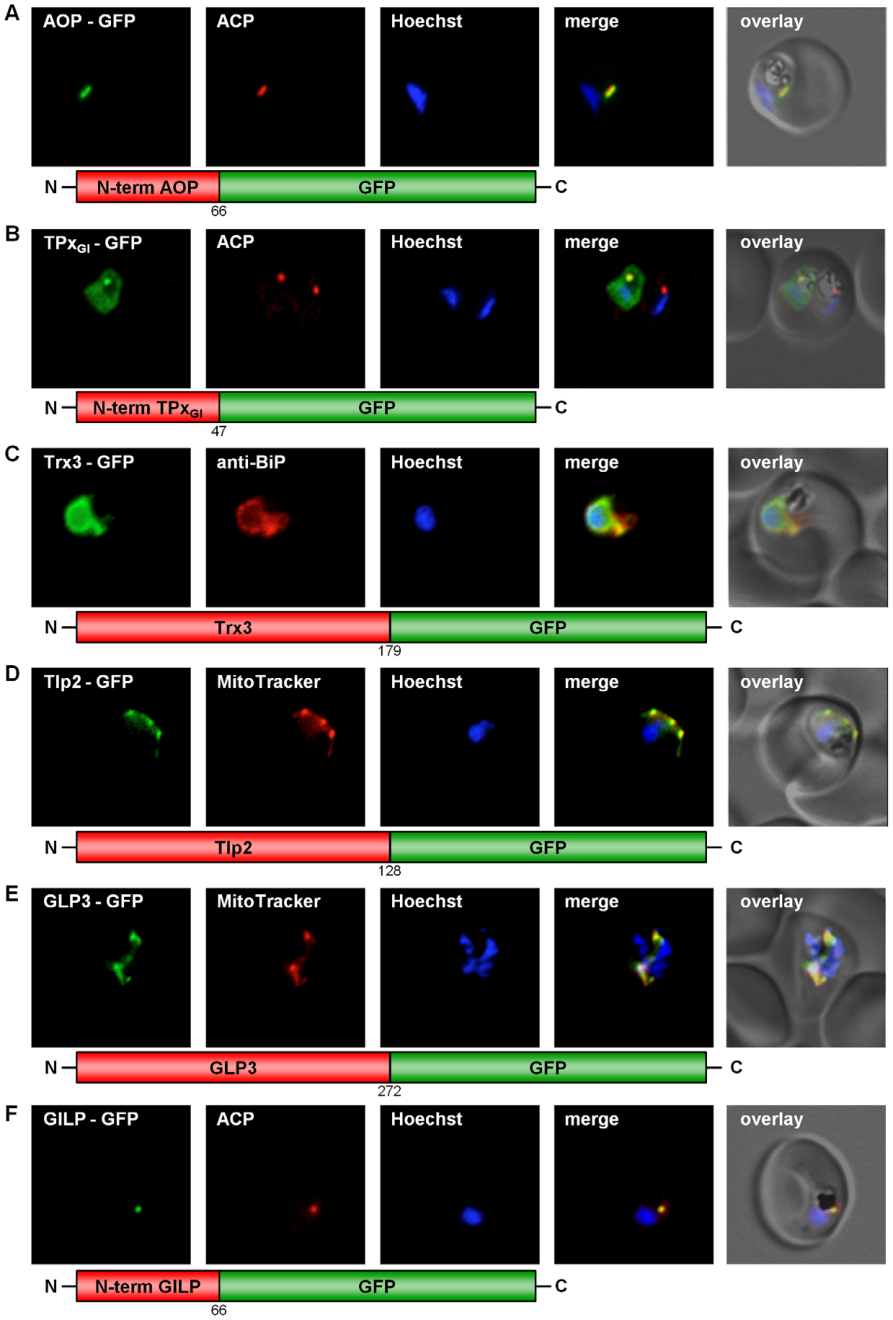
GFP targeting by various *P. falciparum* redox proteins. (A) Apicoplast targeting of the AOP N-terminus. (B) Dual localization (cytosol and apicoplast) of the TPx_Gl_ N-terminal amino acid sequence. (C) ER-targeting of Trx3. (D) Mitochondrial targeting of Tlp2. (E) Mitochondrial targeting of GLP3. (F) Apicoplast targeting of the GILP N-terminal sequence. Colocalization of GFP with the mitochondrial dye MitoTrackerOrange in fixed cells. Colocalization of GFP and the apicoplast marker ACP or the ER marker BiP in fixed, immunodecorated cells.

Four classical Prxs have been identified in *P. falciparum*: the thioredoxin peroxidases 1 and 2 (TPx1, TPx2) [9], [20], a 1-Cys peroxiredoxin (1Cys-Prx) [11], and the so-called antioxidant protein (AOP) [9], [21]. In addition, a glutathione peroxidase-like thioredoxin peroxidase (TPx_Gl_) [22] has been characterised. As described in Table S2, we studied the subcellular localization of all five peroxidases, even when no targeting signal was predicted. For the so far unmapped AOP [8], [21] and TPx_Gl_ we fused the respective N-termini to GFP, and for 1-Cys Prx as well as for TPx1 and 2 we used the complete gene sequence to generate the chimeric construct. Expression of GFP-tagged TPx1 and 1-Cys peroxiredoxin in erythrocytic stages of *P. falciparum* clearly showed that both proteins are cytosolic (Fig. S1A, C). TPx2-GFP expression indicated a localization of TPx2 in the parasite's mitochondrion, as clearly demonstrated by MitoTracker staining (Fig. S1B). This result confirms previously reported data [20]. Furthermore, the AOP-GFP chimera colocalized perfectly with the acyl-carrier-protein (ACP) [23], an established apicoplast marker (Fig. 1A), confirming previous predictions [21]. TPx_Gl_-GFP localized to both the parasitic cytosol and the apicoplast, an evolutionary homologue of the plant chloroplast [24] (Fig. 1B). The specificity of the dual localization to apicoplast and cytosol is evidenced by the lack of cytosolic fluorescence in a non-expressing parasite (Fig. 1B).

### Mapping the subcellular localization of *P. falciparum* thioredoxins and thioredoxin-like proteins

*P. falciparum* possesses at least two classical thioredoxins: Trx1 and Trx2. A third thioredoxin (Trx3) and two thioredoxin-like proteins (Tlp1 and Tlp2) have been characterized [9], [10]. Trx2 appears to have a mitochondrial isoform [20], however strong evidence has been provided that Trx2 is also targeted to the parasitophorous vacuole [25]. We analyzed the subcellular localization of Trx1 and Trx3 as well as of Tlp1 and Tlp2, and studied the localization of Trx2 in more detail (Table S2). Trx1-GFP and Tlp1-GFP (Table S2) localized to the cytosol (Fig. S2A, B). Two chimeric Trx2-GFP-fusion constructs were cloned (Table S2), and their expression in erythrocytic stages of *P. falciparum* led to a clear localization of Trx2 in the parasitophorous vacuole (PV), the compartment separating the parasite from the surrounding host erythrocyte (Fig. S3A). Targeting of Trx2-GFP to the PV was confirmed by subcellular fractionation and analysis of the resulting protein fractions by Western blot [26], also showing Trx2-GFP to be directed to both the PV (lysate fraction) and the parasite (parasite fraction) (Fig. S3D). As controls for the fractionation, we investigated the distribution of PfSERP and PfHsp70. PfSERP is a soluble protein of the PV and was detected in the fraction containing the soluble contents of the PV (lysate fraction in Fig. S3D). PfHsp70 served as indicator for the recovery of intact parasites, marking the parasite cytosol fraction (parasite fraction in Fig. S3D).

In addition to the PV fluorescence, Trx2-GFP labeled further structures within the parasite (Fig. S3B, C) in >90% of the parasites. In these parasites, the GFP fluorescence did neither colocalize with the ER nor with the nucleus, the mitochondrion or the apicoplast, as evidenced by analysis using organellar markers (Fig. S3B, C). In early ring stage parasites, fluorescence was confined to the nascent PV (Fig. S4A). Upon reaching the early trophozoite stage, typically two fluorescent points could be observed, usually lying directly under the parasite plasma membrane (Fig. S4B). With progression through the lifecycle, the number of fluorescent points increased (Fig. S4C, D). In schizont stage parasites, generally numerous fluorescent points were observed (Fig. S4D), however parasites were also observed with only a small number of detectable fluorescent structures (Fig. S4E). The increase of fluorescent foci does not appear to coincide with division of either the mitochondrion or the apicoplast, as even early trophozoite stage parasites could be seen to contain multiple fluorescent structures. Furthermore, the multiple fluorescent points did not appear to associate with single nuclei in schizont stage parasites, suggesting that division does not take place as part of general schizogony and formation of new merozoites. Lastly, this structure does not appear to divide by “branching” akin to the apicoplast and mitochondria, as at no time could a branched form be observed. To support the finding that Trx2-GFP localizes to an organelle, we carried out differential permeabilization of the parasite's cell and organellar membranes followed by a thermolysin protection assay (Fig. S3E) [27]. Low concentrations of digitonin permeabilize the plasma membrane of *P. falciparum* but not organellar membranes, whereas Triton X-100 permeabilizes both the plasma membrane and organellar membranes [27]. If the protease thermolysin is added to digitonin permeabilized parasites, it will cleave all cytosolic proteins, but it cannot act on proteins still protected by intact organellar membranes. If thermolysin is added to Triton X-100 permeabilized parasites, all proteins will be degraded. Protection of both Trx2-GFP and apicoplast-targeted sCdc48 (control) [28] from thermolysin cleavage in digitonin permeabilized parasites is indicative of localization of Trx2-GFP to an organelle (Fig. S3E). Both Trx2-GFP and sCdc48 [28] were completely degraded by thermolysin in cells treated with Triton X-100 (Fig. S3E). The cytosolic control tubulin was degraded by thermolysin in both digitonin and Triton X-100 treated cells, but remained intact when cells were permeabilized with digitonin in the presence of EDTA (inhibitor of thermolysin) (Fig. S3E). Further analysis of this novel, non-dividing, Trx2 containing compartment is currently being carried out.

Both Trx3-GFP chimeras (Table S2) were unambiguously targeted to the endoplasmic reticulum (Fig. 1C). Because the green signal of the GFP-tagged Trx3 protein is slightly stronger than the red signal of the ER protein BiP, the yellow colocalization signal may appear weak (Fig. 1C). However, when comparing the pattern of fluorescence it becomes evident that both signals originate from the same structure (Fig. 1C). This result is further substantiated by the prediction of a transmembrane helix in the N-terminus of Trx3 (Table S2) [29]. In the case of Tlp2-GFP a clear mitochondrial targeting could be shown (Fig. 1D).

### Mapping the subcellular localization of *P. falciparum* glutaredoxin-like proteins

Glutaredoxin-like proteins (GLPs) form a new subgroup of glutaredoxins with a serine replacing the second cysteine in the CxxC-motif of the active site [5], [6]. In *P. falciparum* one classical Grx, and three glutaredoxin-like proteins (GLP1-3) [5], [6] have been identified. In order to study the subcellular localization of the three *P. falciparum* GLPs *in vivo*, three full length constructs fused to GFP were generated (Table S2) and transfected into *P. falciparum* blood stage parasites. Analysis of GLP1-GFP and GLP2-GFP clearly demonstrated a cytosolic localization for these proteins (Fig. S5A, B). GLP3-GFP, however, was targeted to the mitochondrion, as demonstrated by its colocalization with MitoTracker (Fig. 1E).

### Mapping the subcellular localization of a second putative *P. falciparum* glyoxalase system

*P. falciparum* possesses a cytosolic glyoxalase system, comprising cytosolic glyoxalase 1 and 2 [8], [30]. However, a glyoxalase-1-like-protein (GILP) and a second glyoxalase 2 with a targeting sequence (tGloII) were also identified [8], [30]. In our studies GILP-GFP showed colocalization with the acyl-carrier-protein (ACP) [23], a marker for the apicoplast (Fig. 1F). The apicoplast localization reported previously for tGloII-GFP [30] was unambiguously reproduced in our experimental approach (Fig. S5C). In addition tGloII-GFP was found to localize to the cytosol (Fig. S5C).

### PfGR and PfTrxR have a putative second start codon

Since the genome sequence of *P. falciparum* [31] has become available, *in silico* analysis of both the Pf*trxr* (accession number CAA60574) gene locus and the Pf*gr* (accession number CAA63747) gene locus in our laboratory revealed a second putative start codon upstream of the predicted start (Supporting Information S1). The resulting additional sequence fragments comprise 228 bp and 138 bp, respectively (Supporting Information S1) [9]. These alternative starts do not involve an additional intron, and the 5′-elongations were confirmed experimentally via isolation from a *c*DNA library. In the case of TrxR, the respective alternative recombinant enzyme (TrxR; accession number AAQ07981) was biochemically characterized and found to be very similar in substrate specificity and both kinetic and biochemical behavior to the previously studied shorter variant [9], [32]. Interestingly, this new N-terminal sequence is not predicted as a target sequence (Table S2). In contrast, GR exhibits an N-terminal extension that consists of a putative signal peptide (SP) followed by a hydrophilic section with a net positive charge, thus showing the essential characteristics of bipartite topogenic signals (BTS) that commonly direct proteins to the apicoplast (Table S2) [33], [34].

### Alternative translation initiation of PfGR

**Figure 2:**
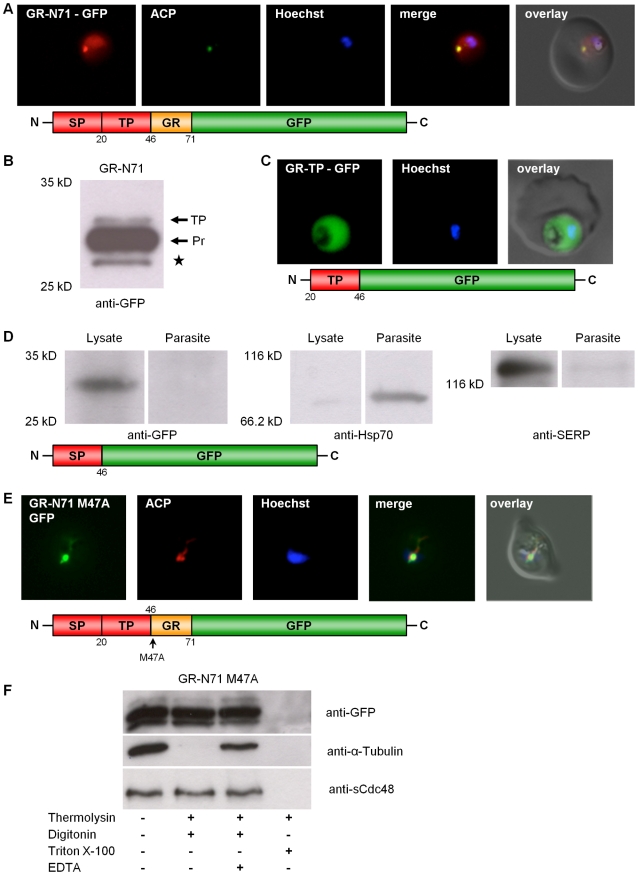
Dissection of the dual localization of *P. falciparum* GR. (A) Dual localization (cytosol and apicoplast) of GR-N71-GFP effected by a newly discovered N-terminal extension/leader. (B) Western blot analysis of parasites stably expressing GR-N71-GFP using anti-GFP antibodies. TP, transit peptide still attached; Pr, transit peptide processed; **★**, GFP degradation product. (C) Cytosolic targeting of a construct lacking the signal peptide (GR-TP-GFP). (D) Western blot analysis of parasites stably expressing GR-SP using anti-GFP, anti-Hsp70, and anti-SERP antibodies shows targeting of GR-SP to the parasitophorous vacuole. Lysate, erythrocyte cytosol plus the soluble contents of the parasitophorous vacuole; Parasite, cellular contents of the parasite. (E) Apicoplast targeting of construct (A) after mutation of methionine 47 to alanine (GR-N71-GFP M47A). (F) Thermolysin protection assays on parasites stably expressing GR-N71-GFP M47A confirm complete apicoplast localization of GR-N71 M47A. Parasites permeabilized using the detergents digitonin (plasma membrane) and Triton X-100 (plasma membrane and organellar membranes) were treated with the protease thermolysin. Tubulin is the cytosolic control that is not protected from thermolysin after digitonin permeabilization; sCdc48 is the apicoplast-targeted control protein that is protected from thermolysin after digitonin permeabilization but not after Triton X-100 permeabilization. Degradation could be inhibited with the addition of EDTA, an inhibitor of thermolysin, suggesting that the loss of protein we observed was specifically due to thermolysin degradation. Colocalization of GFP and the apicoplast marker ACP in fixed, immunodecorated cells. Live cell imaging of erythrocytes infected with transgenic parasites for solely cytosolic GFP signals.

In order to study the subcellular localization of PfGR *in vivo*, a construct comprising the first 213 bp (composed of 138 bp of the 5′-elongation plus 75 bp of the previously known GR) fused to the GFP-gene was generated and transfected into *P. falciparum* blood stage parasites. This C-terminally GFP-tagged GR construct (GR-N71-GFP) was directed to both the parasitic cytosol and the apicoplast (Fig. 2A) as shown by colocalization with ACP [23]. The majority of apicoplast proteins are nuclear encoded. They are targeted to the apicoplast via a bipartite topogenic signal (BTS) at the N-terminus of the protein. This signal is composed of a signal peptide (SP), responsible for targeting the pre-proteins into the secretory pathway, followed by a plant-like transit peptide (TP) that directs the pre-proteins to the apicoplast [23], [33], [34], [35]. Both portions of the BTS are usually proteolytically processed during traffic to the apicoplast: The SP of the bipartite targeting sequence is cleaved during translocation into the ER, and the TP becomes cleaved upon import into the apicoplast, resulting in the mature protein. As SP processing is a rapid event, pre-proteins containing this peptide are usually not detectable by Western blot [36]. We hypothesized that, if GR contains a weak signal peptide, this may result in only a portion of the nascent protein population being captured by the signal-recognition-particle and directed to the ER for translocation and SP cleavage. This would leave the other portion as a cytosolic protein. To investigate this, we studied processing of the GR-N71-GFP fusion protein by Western blot. Using anti-GFP antibodies (Roche) and total cell lysates from stably transfected parasites, we were able to detect (i) the TP bearing precursor protein (app. 33 kDa), where the SP was already proteolytically removed, and (ii) the completely processed protein (app. 30 kDa), where also the TP was cleaved (Fig. 2B). The band around 28 kDa corresponds to the previously described GFP-degradation product (Fig. 2B) [34]. Interestingly, no full length pre-protein (bearing both the SP and the TP) could be detected, which contradicts the weak SP hypothesis. To further define the sequence information responsible for apicoplast targeting of GR-N71-GFP, we generated two chimeric constructs with either the signal peptide (GR-SP) or the transit peptide (GR-TP) fused to GFP. Consistent with the two-step mechanism of plastid targeting in apicomplexan parasites, signal peptide deletion (GR-TP-GFP) from the targeting sequence resulted in cytosolic GFP accumulation (Fig. 2C) [33], [34]. In the case of the parasites stably transfected with the GR-SP-GFP construct, GFP localized predominantly to the parasitophorous vacuole (Fig. S5D). This result was confirmed by subcellular fractionation and analysis of the resulting protein fractions by Western blot [26] as described for Trx2, also showing GR-SP to be directed to the parasitophorous vacuole (lysate) (Fig. 2D). This result verified the functionality of the signal peptide of GR [33], [34].

**Figure 3:**
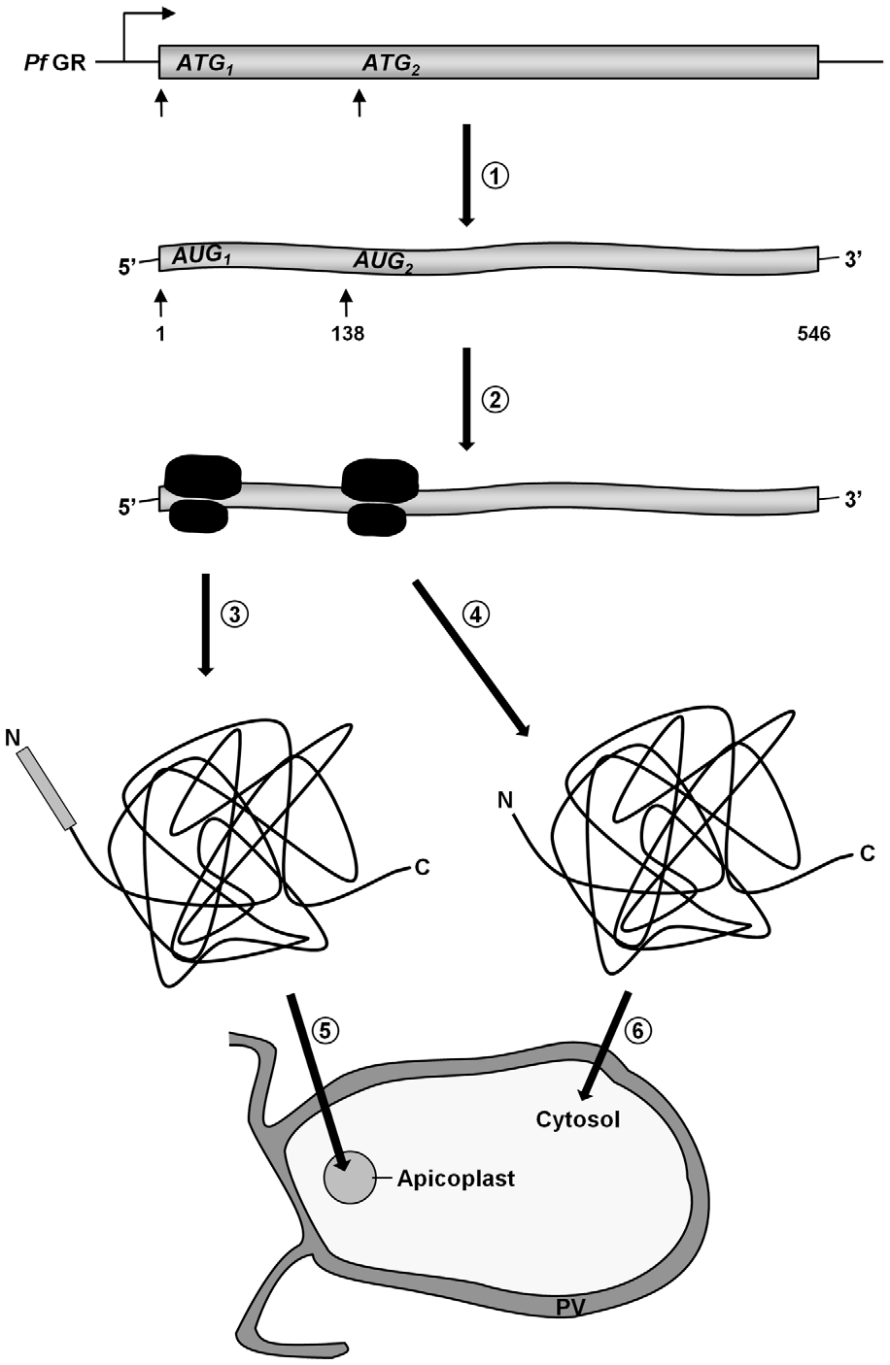
A schematic representation of GR distribution in *P. falciparum* by ATI. (1) A single gene is transcribed, leading to a single transcript. (2) An upstream translational start site followed by a second AUG-codon, leading to two translational products. (3) GR carrying an N-terminal apicoplast targeting sequence. (4) GR lacking an N-terminal targeting sequence. (5) GR translocates into the apicoplast. (6) GR remains in the cytosol. PV, parasitophorous vacuole.

Based on (a) the fact that we were able to clone the 5′-elongated form of GR from *c*DNA, together with (b) the presence of two possible start-ATG codons flanking the BTS, as well as (c) the functionality of the GR-SP, we proposed alternative-translation-initiation (ATI) [37], [38], [39] as an explanation for the two subcellular localizations of PfGR. According to current knowledge on translation initiation mechanisms, eukaryotic ribosomes can recognize several alternative translation start sites and the number of experimentally verified examples of ATI is growing rapidly [37], [38], [39]. Therefore ATI is a molecular mechanism potentially allowing a single mRNA to produce several protein-isoforms. To verify this hypothesis, we mutated the methionine of the second start codon to alanine in the GR-N71-GFP construct (GR-N71-GFP M47A). IFA on stably transfected blood stage parasites with ACP [23] showed an exclusive apicoplast localization of GR-N71-GFP M47A (Fig. 2E). To further support apicoplast targeting of GR-N71-GFP M47A, we performed differential permeabilization of the parasite's cell and organellar membranes followed by a thermolysin protection assay (Fig. 2F) [27], as described for Trx2. Protection of GR-N71-GFP M47A from thermolysin cleavage in digitonin permeabilized parasites proves localization of GR-N71-GFP M47A to the apicoplast. These results confirm that ATI leads to the dual localization of GR. The whole process is shown schematically in Figure 3.

### Alternative-translation initiation of PfTrxR

**Figure 4:**
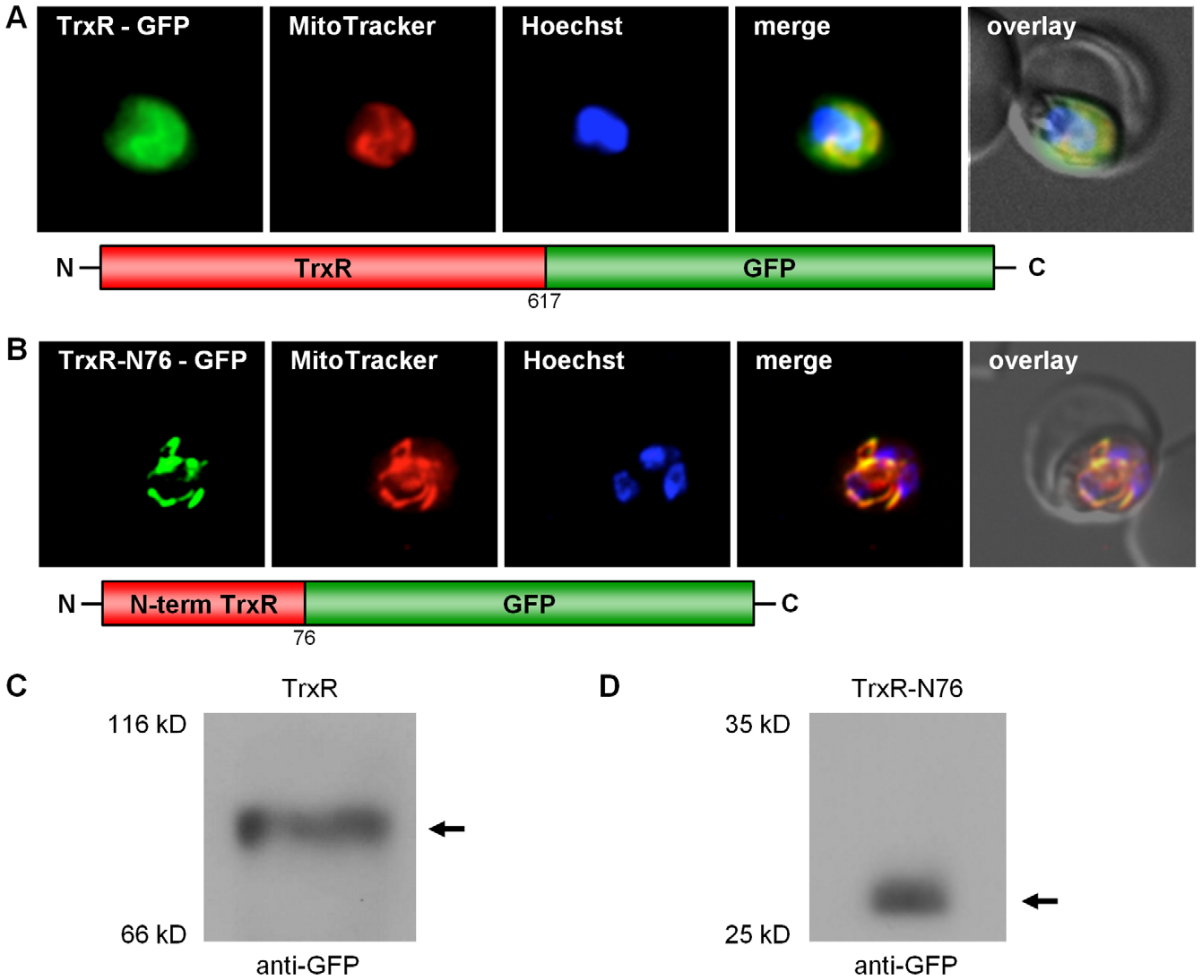
Dissection of the dual localization of *P. falciparum* TrxR. (A) Dual localization (cytosol and mitochondria) of TrxR-GFP effected by a newly discovered N-terminal extension/leader. (B) Mitochondrial targeting of a construct (TrxR-N76-GFP) containing solely the TrxR 5′-extension. (C) Detection of TrxR-GFP fusion constructs in transgenic parasites by Western blot analysis against the GFP-moiety, showing that the dual localization of TrxR-GFP is not due to a weak mitochondrial signal sequence. (D) Western blot analysis of parasites stably expressing TrxR-N76-GFP using anti-GFP antibodies, showing proteolytically processed TrxR. Colocalization of GFP with the mitochondrial dye MitoTrackerOrange in fixed cells.

As previously mentioned, the N-terminal sequence extension discovered for PfTrxR was not predicted to be a target sequence (Table S2). In this context, it is worth mentioning that *Drosophila melanogaster* TrxR also contains an unusual N-terminal elongation, which is not predicted to have a targeting function. For this enzyme, however, transport into the mitochondrion has been demonstrated experimentally [40]. Furthermore, existing algorithms do not always correctly predict targeting signals in *Plasmodium* redox active proteins, as shown for e.g. *P. falciparum* mitochondrial SOD [13]. In order to study the subcellular localization of the 5′-elongated TrxR *in vivo*, a construct of the full length TrxR fused to the GFP gene (TrxR-GFP) was generated. In addition to the cytosol, TrxR-GFP clearly located to the mitochondrion (Fig. 4A). The dual localization of the alternative form of TrxR can have two reasons: (i) the target peptide responsible for post-translational import of TrxR into the mitochondrion represents a weak signal and therefore a part of the synthesized protein is not recognized by the mitochondrial import machinery; or (ii) the second ATG, previously predicted to be the initiation codon, gives rise to an ATI-site [37], [38], similar to GR. To answer this question, we studied the processing of the TrxR-GFP fusion protein by Western blot using anti-GFP antibodies (Roche) and total cell lysates from stably transfected parasites. In the case of a weak mitochondrial targeting sequence, we would expect to see two strong bands, one band around 97 kDa being the unprocessed cytosolic precursor protein not imported into the mitochondrion, and a second band around 88 kDa representing the mitochondrial-imported and proteolytically processed protein. In the case of an ATI-site [37], [38], [39] we would expect to see one strong band around 88 kDa, representing both the cytosolic and the mitochondrial-imported protein. The Western blot for the TrxR-GFP construct clearly showed one band around 88 kDa, which strongly indicates that the dual localization of TrxR is based on ATI (Fig. 4C). To further support our hypothesis, we transfected blood stage parasites with a construct that only contained the 5′-extension of TrxR fused to the GFP gene (TrxR-N76-GFP), without the second ATG start-codon. TrxR-N76-GFP localized exclusively to the mitochondrion, and the Western blot showed one band depicting the proteolytically processed form of the protein (Fig. 4B, D).

## Discussion

**Figure 5:**
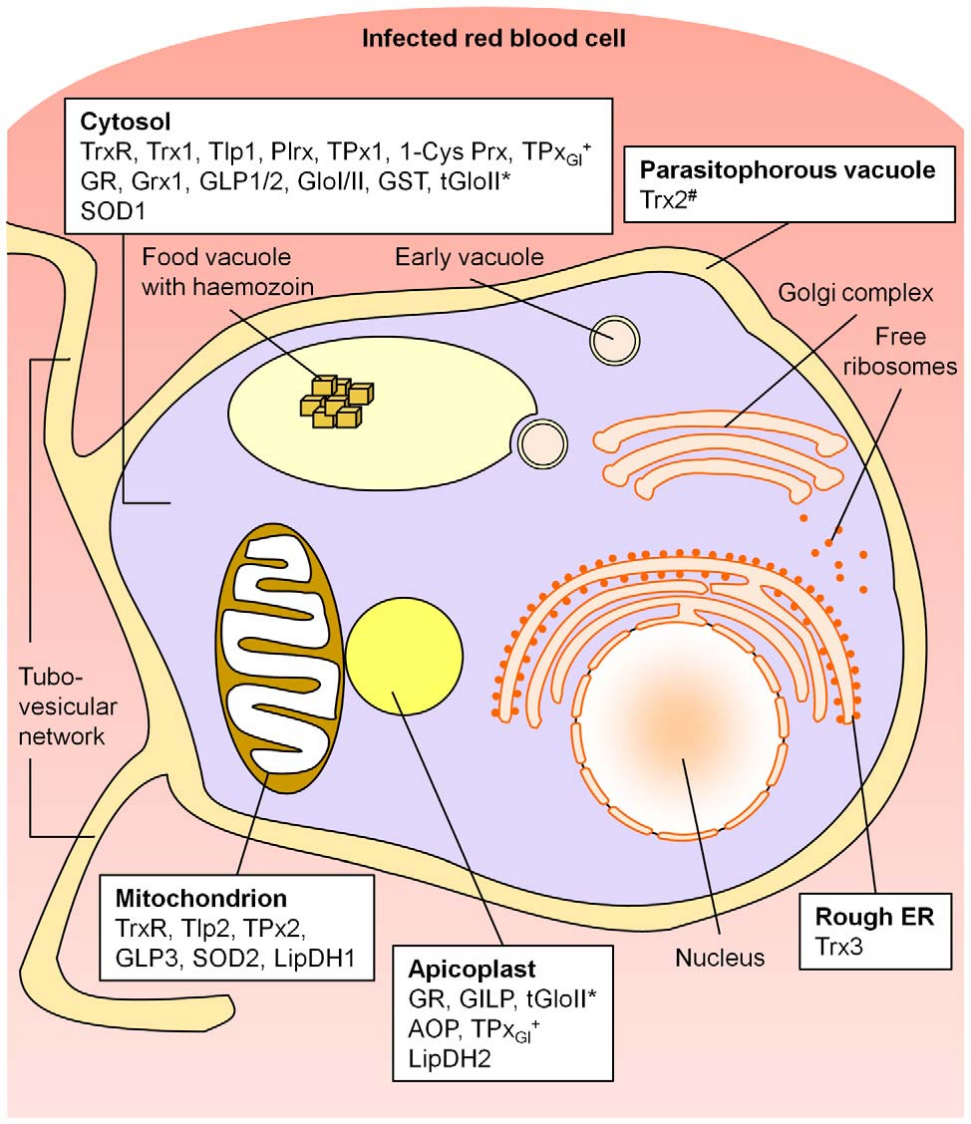
Compartmentation of the redox metabolism in malaria parasites. Schematic representation of an intra-erythrocytic trophozoite, highlighting key parasite intracellular compartments. AOP, antioxidant protein; 1-Cys Prx, 1-cysteine peroxiredoxin; ER, endoplasmic reticulum; GILP, glyoxalase-1-like protein; Glo, glyoxalase; GLP, glutaredoxin-like protein; GR, glutathione reductase; Grx, glutaredoxin; GST, glutathione-S-transferase; LipDH, lipoamide dehydrogenase-like protein; Plrx, plasmoredoxin; SOD, superoxide dismutase; Tlp, thioredoxin-like protein; TPx, thioredoxin-dependent peroxidase; Trx, thioredoxin; TrxR, thioredoxin reductase. *, tGloII was found to localize to the cytosol and the apicoplast; ^#^, Trx2 was targeted to the parasitophorous vacuole and a yet unidentified organelle of the parasite; ^+^, TPx_Gl_ was localized both to the cytosol and the apicoplast (targeting mechanism not yet analyzed). Please refer to the main text for further details and references. Endogenous glutathione peroxidases and a catalase are not present in *Plasmodium*.

Our study provides the first comprehensive picture of the subcellular compartmentation of cellular redox metabolism in malaria parasites. An overview of the data is given in Figure 5.

The apicoplast, a non-photosynthetic plastid of *Plasmodium*, homologous to chloroplasts of plants, harbours indispensable plant-like metabolic pathways including fatty-acid and haem biosynthesis [24]. Until today no extensive data existed about the antioxidative capacity of the apicoplast. Based on our data, five directly redox-related enzymes are targeted to this apicomplexan-specific organelle (see Fig. 5). AOP [9], [21] and TPx_Gl_ [2], [22] belong to the family of peroxiredoxins, which are important components of the cellular antioxidative and redox-regulatory systems (for TPx_Gl_ see also cytosol). PfAOP is one of the few AOPs reported in a non-plant organism and likely to be the only Prx in *P. falciparum* that turns over lipid hydroperoxides [9], [21]. We could also show that, apart from a cytosolic glyoxalase couple, two other glyoxalase related proteins, GILP [8] and tGloII [8], [30], are transported to the apicoplast. It is known that the apicoplast imports triosephosphate-isomerase [24], [41] and its substrates and that one of the sources of toxic methylglyoxal is an incomplete triosephosphate-isomerase reaction [8], [24]. Therefore, an apicoplast-glyoxalase-system to detoxify methylglyoxal and other 2-oxoaldehydes would have a functional importance. The existence of a functional GSH-dependent glyoxalase system in the apicoplast is further supported by our finding that a previously unidentified isoform of PfGR is targeted to the apicoplast (Fig. 2). GR is a single copy gene within the *Plasmodium* genome, and the apicoplast isoform is generated by alternative-translation-initiation (ATI) at a start-codon upstream of the canonical start (schematically depicted in Fig. 3). The expression of the cytosolic GR is strictly dependent on the integrity of the downstream AUG triplet as shown by *in vitro* and *in vivo* functional analysis of mutants. This indicates that the phenomenon is a direct consequence of the translational process, supporting the leaky scanning model for ATI [37], [38]. In conclusion, the discovery of an antioxidative and redox-regulatory system within the apicoplast fills an important gap in our knowledge about this apicomplexan-specific organelle and has an impact on the interpretation of other studies [24], [42]. For example, the plastidic-type ferredoxin and ferredoxin-NADP^+^ reductase (FNR) of *P. falciparum* provide electrons for the synthesis of isoprenoid precursors in the apicoplast [42]. FNR activity is controlled through monomer-dimer interconversion by oxidizing and reducing agents, such as H_2_O_2_ and glutathione [42]. Furthermore, our data represent a solid basis for detailed functional analyzes.

The mitochondrial antioxidant and redox-regulatory proteins of *Plasmodium* proposed so far comprise Trx2 [20] and TPx2 [20] as well as a mitochondrial SOD [13] and a dihydrolipoamide dehydrogenase [14]. The assumption that mitochondrial TrxR does not exist in malarial parasites led to the postulation that the low-M_r_ thiol lipoic acid, an essential cofactor of mitochondrial α-ketoacid dehydrogenase complexes, acts as a reductant for PfTrx2 *in vivo* [20]. Here we describe a previously unidentified isoform of PfTrxR localized to the mitochondrion (Fig. 4), proposing a disulfide-reductase based reduction system. As with GR, TrxR is a single copy gene within the *Plasmodium* genome and the mitochondrion targeted isoform is generated by an ATI-site upstream of the canonical start. We did not detect mitochondrial targeting for Trx2 [20], however its localization to the parasitophorous vacuole could be confirmed [25]. Furthermore, as described in detail in the results section, we found Trx2 localizing to a yet unidentified, non-dividing organelle of the parasite (Fig. S3, Fig. S4). For Tlp2, targeting to the mitochondrion could be shown unambiguously (Fig. 1D), and mitochondrial targeting of TPx2 could be confirmed (Fig. S1B). Therefore, the existence of a mitochondrial thioredoxin-dependent antioxidative network in *P. falciparum* can be postulated. Additionally, we demonstrated that GLP3 is a mitochondrial protein, despite a lack of predictable protein targeting signals (Table S2; Fig. 1E).

The function of Trx2 in the parasitophorous vacuole was recently unraveled [25]. Trx2 appears to be part of a newly discovered export machinery of the malaria parasite in the parasitophorous vacuolar membrane and is potentially involved in unfolding of target proteins for their passage through the export channel [25].

Within the frame of this study we identified a thioredoxin targeted to the ER (Fig. 1C). The localization of Trx3 to the ER might indicate a specialized function with an involvement in protein folding through the formation and isomerization of disulfide bonds [43].

The cytosol harbors the major part of the redox-capacity and antioxidative potential of the parasite (Fig. 5). Apart from the previously known cytosolic components, our study unravels further important details of this system. Within the glutathione-system of *P. falciparum*, GLP 1 and 2 were shown to be cytosolic proteins, although GLP1 was predicted to possess an N-terminal mitochondrial targeting sequence [5], [6] (Fig. S5A, B). Especially intriguing in this light is a recent report by McFadden and coworkers, who could demonstrate that exon 1 of GLP1 perfectly fulfils the criteria of a transit peptide for apicoplast targeting. When placed between a signal peptide and GFP, exon 1 of GLP1 mediates accurate plastid targeting [35]. The targeting mechanism of TPx_Gl_, a Prx, both to the cytosol and to the apicoplast has not yet been analyzed in further detail (Fig. 1B). Considering that the gene sequence contains two additional ATG-codons within the target sequence, we propose that TPx_Gl_ is targeted to both compartments due to ATI [37], [38], as we have shown here for GR and TrxR.

In the present study we mapped the cellular redox system of malaria parasites, providing a tool to better understand the cellular functions and interactions of this essential network. Furthermore, we demonstrated that ATI exists in apicomplexan parasites and is used to translate protein isoforms with differing subcellular localization from one gene. The differentiated control of ATI-sites may furthermore provide a mechanism for the fine tuning of the ratio between the synthesized protein isoforms. In fact, reports of dual/multiple targeting [39], [44] in apicomplexan parasites exist where ATI could be a possible explanation for the described results. Identification of the genes that might have evolved dual targeting capability because of ATI-sites is likely to change our *in silico* view of organelle function.

## Methods

### Construction of *P. falciparum* expression plasmids

All primers used to clone or mutate the described constructs are given in Table S3. Furthermore, all proteins (with one exception: alternative, 5′-elongated GR) described in this study are available in our laboratory. The 5′elongated form of the GR-gene was cloned from *c*DNA. For GFP-fusion constructs standard PCR amplified regions of the respective genes were cloned in front of the GFP gene using the pSK-vector (Stratagene) and *Bgl*II and *Avr*II restriction sites (Fermentas). After sequencing, GFP fusion constructs were subcloned into pARL-1a+ (kindly provided by Prof. Cowman, Melbourne, and colleagues) with *Xho*I (Fermentas).

### Parasite culture and transfection

Blood stages of *P. falciparum* strain 3D7 were maintained in continuous culture as described previously [45]. Transfection with the pARL-1a+ constructs was carried out by electroporation and drug selection using 2 nM of WR99210 (kindly supplied by D. Jacobus, Princeton, New Jersey, USA) as previously described [46].

### Live cell imaging and immunofluorescence assays

For live cell imaging parasites were stained with Hoechst 33258 (10 µg/ml) and directly imaged at room temperature for not longer than 20 min. Images were acquired using a Zeiss Axio Observer inverse epifluorescence microscope system with appropriate filter sets, equipped with Axiovision 4 software. Exposure times were chosen to minimize bleaching but allow visualization of details. Immunofluorescence experiments were carried out by fixing the cells with 4% paraformaldehyde/0.0075% glutaraldehyde in PBS pH 7.4 as described previously [47] except that fixation was carried out for 30 min at 37°C and quenching was in 100 mM glycine/PBS. The following primary antibodies were used: rabbit anti-ACP (1∶500; kindly provided by G. McFadden, Melbourne), rabbit anti-BiP (1∶2,200; kindly provided by T. Gilberger, Hamburg), chicken anti-GFP (1∶1,000; Abcam). Suitable Cy2-Cy3- (Dianova) conjugated secondary antibodies were used 1∶2,000. Antibodies were diluted in 3% bovine serum albumin-PBS. MitoTrackerOrange (Molecular Probes) was used at 20 nM. Fixed parasites were co-stained with Hoechst 33258 (50 ng/ml) to visualize nuclear DNA. All data shown are representative of at least 20 independent observations.

### Image processing and presentation

Individual images were imported into Image J64 (version 1.43b, available at http://rsb.info.nih.gov/ij), converted to 8-bit grayscale, subjected to background subtraction, and overlaid. Pictures were adjusted to gain optimal contrast to visualize features of interest. To create figures, TIF files were imported into MS-PowerPoint, false colored, assembled, and slides were exported as TIFs. No gamma adjustments were applied to any images, and all data are presented in accordance with the recommendations of Rossner and Yamada [48].

### Western blotting

The method to separate the parasitophorous vacuole from the parasite was described previously [26]. Briefly, trophozoite infected red blood cells (IRBC), 24–30 h postinfection, were enriched using LD-columns (MACS, Miltenyi Biotec) [49]. IRBC were harvested at 300 g for 3 min at room temperature. IRBC (in aliquots of 2×10^8^ cells) were incubated in 200 µL of 0.1% saponin in PBS pH 7.4 at room temperature for 1 min. Then the sample was centrifuged at 1,500 g for 3 min. The supernatant, containing the erythrocyte cytosol plus the soluble contents of the PV, was removed carefully and centrifuged at 35,000 g for 30 min at 4°C to remove cell debris. The parasite pellet, containing the intact parasite devoid of the host cytosol and PV contents, was washed 3 times with 200 µL of PBS containing protease inhibitors (Pefablock, Roth; Complete, Roche) and harvested at 1,500 g for 5 min. Both the parasite pellet and the supernatant containing the erythrocyte cytosol plus the soluble contents of the PV were separated on SDS-gels corresponding to 5×10^6^–1×10^7^ parasites/lane and transferred to a PVDF membrane (Roth). Membranes were probed with anti-GFP (1∶1,000; Roche), anti-Hsp70 (1∶1,000; kindly provided by T. Blisnick, Paris), and anti-SERP (1∶500; kindly provided by K. Lingelbach, Marburg) antibodies, followed by HRP-conjugated secondary antibodies (1∶10,000; Jackson ImmunoResearch). All antibodies for Western blot were diluted in 5% non-fat milk in TBST.

For Western blot analysis of total parasite cell lysates, parasites were treated equally. Differences were that: (i) enriched IRBC were incubated with 0.1% saponin in PBS pH 7.4 at room temperature for 30–40 sec, (ii) only the parasitic pellet was kept for further analysis, and (iii) membranes were probed only with an anti-GFP antibody (1∶1,000; Roche).

### Thermolysin protection assay

The thermolysin protection assay was carried out as described previously [27] with minor changes. Briefly, trophozoite infected red blood cells were enriched and harvested as described for the parasitophorous vacuole preparation. Parasites released from RBCs by saponin lysis were resuspended in assay buffer (50 mM HEPES-NaOH, pH 7.4, 0.5 mM CaCl_2_, 300 mM sorbitol) containing no detergents (control), 0.05% digitonin, 0.05% digitonin +10 mM EDTA or 1% Triton X-100. After incubation at 25°C for 10 min, thermolysin (Fluka) was added to a final concentration of 25 µg per 1 mg of parasite protein. Reactions were incubated at 25°C for 20 min. The reactions were stopped by the addition of 10 mM EDTA. Proteins were precipitated using trichloracetic acid and protein pellets were washed with cold acetone. Proteins were separated by SDS-PAGE and subjected to Western blotting. Membranes were probed with anti-GFP (1∶1,000; Roche), anti-sCdc48 (1∶1,000) [28], and anti-Tubulin (1∶1000; Sigma) antibodies, followed by HRP-conjugated secondary antibodies (1∶10,000; Jackson ImmunoResearch). All antibodies for Western blot were diluted in 5% non-fat milk in TBST.

## Supporting Information

Supporting Information S1 Shown here is the genomic sequence of PfTrxR containing the alternative start 5′ of the previously predicted start and the genomic sequence of PfGR containing the first exon with the alternative start 5′ of the previously predicted start.
(0.03 MB DOC)

Table S1 Accession numbers of redox-related proteins.
(0.04 MB DOC)

Table S2 Overview of the analyzed redox proteins.
(0.18 MB DOC)

Table S3 Oligonucleotide primers.
(0.10 MB DOC)

Figure S1 GFP targeting by various *P. falciparum* peroxiredoxins. (A) Cytosolic localization of TPx1. (B) Mitochondrial targeting of TPx2. (C) Cytosolic localization of 1-Cys Prx. Live cell imaging of erythrocytes infected with transgenic parasites for solely cytosolic GFP signals. Colocalization of GFP with the mitochondrial stain MitoTrackerOrange in fixed cells.
(0.63 MB TIF)

Figure S2 GFP targeting by *P. falciparum* thioredoxin 1 and thioredoxin-like protein 1. (A) Cytosolic localization of Trx1. (B) Cytosolic localization of Tlp1. Live cell imaging of erythrocytes infected with transgenic parasites for solely cytosolic GFP signals.
(0.48 MB TIF)

Figure S3 GFP targeting by *P. falciparum* thioredoxin 2. (A)–(C) Targeting of Trx2 to the parasitophorous vacuole and to a not yet characterized, non-dividing organelle within the parasite. (D) Western blot analysis of parasites stably expressing Trx2-GFP using anti-GFP, anti-Hsp70, and anti-SERP antibodies confirms dual localization of Trx2 to the parasitophorous vacuole and the cellular part of parasite. Lysate, erythrocyte cytosol plus the soluble contents of the parasitophorous vacuole; Parasite, cellular contents of the parasite. (E) Thermolysin protection assays on parasites stably expressing Trx2-GFP confirm organellar localization of Trx2. Parasites permeabilized using the detergents digitonin (plasma membrane) and Triton X-100 (plasma membrane and organellar membranes) were treated with the protease thermolysin. Tubulin is the cytosolic control that is not protected from thermolysin after digitonin permeabilization; sCdc48 is the apicoplast-targeted control protein that is protected from thermolysin after dititonin permeabilization but not after Triton X-100 permeabilization. Degradation could be inhibited with the addition of EDTA, an inhibitor of thermolysin, suggesting that the loss of protein we observed was specifically due to thermolysin degradation. Colocalization of GFP with the mitochondrial stain MitoTrackerOrange in fixed cells. Colocalization of GFP and the apicoplast marker ACP in fixed, immunodecorated cells.
(1.72 MB TIF)

Figure S4 GFP targeting by thioredoxin 2 in various stages of *P. falciparum*. (A) Early ring stage parasite showing Trx2-GFP targeting to the PV. (B) Early trophozoite stage parasite showing two fluorescent Trx2-GFP points lying directly under the parasite plasma membrane. (C) Late trophozoite/early schizont stage parasite showing numerous fluorescent Trx2-GFP points. (D) Schizont stage parasite showing numerous fluorescent Trx2-GFP points. (E) Schizont stage parasite showing a small number of observable fluorescent Trx2-GFP structures. Live cell imaging of erythrocytes infected with transgenic parasites.
(0.69 MB TIF)

Figure S5 GFP targeting by various *P. falciparum* redox proteins of the glutathione system. (A) Cytosolic localization of GLP1. (B) Cytosolic localization of GLP2. (C) Dual localization (cytosol and apicoplast) of the tGloII N-terminus. (D) PV-localization of the signal peptide of GR (the parasites showed a fast bleaching fluorescence, leading to a high background signal). Live cell imaging of erythrocytes infected with transgenic parasites for solely cytosolic GFP signals. Colocalization of GFP and the apicoplast marker ACP in fixed, immunodecorated cells.
(0.89 MB TIF)
