# Supplementary material for: Compartmentation of Redox Metabolism in Malaria Parasites
Source: PLoS Pathog. 2010 Dec 23;6(12):e1001242. doi: 10.1371/journal.ppat.1001242 (PMC3009606; doi:10.1371/journal.ppat.1001242)
Supplement: Supporting Information S1 — Shown here is the genomic sequence of PfTrxR containing the alternative start 5′ of the previously predicted start and the genomic sequence of PfGR containing the first exon with the alternative start 5′ of the previously predicted start. (0.03 MB DOC) [file ppat.1001242.s001.doc]

**Supporting Information S1**

Shown here is the genomic sequence of PfTrxR containing the alternative start (boxed M) 5’ of the previously predicted start, which is given in bold. The new start does not involve a splicing event and the respective elongated sequence can now be found in PlasmoDB, PFI1170c, where it replaces the older, shorter version of the TrxR (or GenBank, accno. AAQ07981).

TTACATTAAAATAAAAAAAAAAAAAATAAAATAAAATAAAAATTATGAACAATGTAATTT

T L K * K K K K * N K I K I M N N V I S

CTTTCATTGGAAATTCATCAAATAAATATTTCCAAATTAATCAACTTCATTTTATTAGGA

F I G N S S N K Y F Q I N Q L H F I R I

TCATCAATAAAAATATACATTCTAAGAATAATCTTATTAACTCTAATTCATCTTATAATG

I N K N I H S K N N L I N S N S S Y N V

TTTTTTATAATAAATATTTTATAAAGAACACATTCCAAAATAAAAATAAACTATCCTCCA

F Y N K Y F I K N T F Q N K N K L S S I

TATATTCCAAATTAAACTTTTCCATTAAAAAC**ATGTGTAAAGATAAAAACGAAAAAAAAA**

Y S K L N F S I K N **M C K D K N E K K N ...**

Shown here is the genomic sequence of PfGR containing the first exon with the alternative start (boxed M) 5’ of the previously predicted start, which is given in bold. The new start does not involve an additional splicing event. The respective elongated sequence can now be found in GenBank, accno. HQ399186.

TCATTTTATTTTTTTTTTTTTTTTTTCTTATAAAACTTATTAATTATGTACAAACATAGA

S F Y F F F F F F L * N L L I M Y K H R

TACTTTCATTTTTTTTTCTTTTTCTTTTTTTTTCTCGTGTCAACCAAAATAATAAGAAGT

Y F H F F F F F F F F L V S T K I I R S

TTTACTTTTCTTAACAATAATACAAATTTGAGTAATCCAGTATACTTTAAAAAAAAAGCA

F T F L N N N T N L S N P V Y F K K K A

AATATGGTTTACGATTTAATTGTAATTGGTGGTGGAAGTGGAGGAATGGCTGCAGCTAGG

N **M V Y D L I V I G G G S G G M A A A R**

**Exon 1**

AGGGCAGCAAGGTTATATGAAAATAAATAATAAGTCCATACATATATGTATGTATATATA

**R A A** **R** L Y E N K * * V H T Y M Y V Y I
