# Supplementary material for: Compartmentation of Redox Metabolism in Malaria Parasites
Source: PLoS Pathog. 2010 Dec 23;6(12):e1001242. doi: 10.1371/journal.ppat.1001242 (PMC3009606; doi:10.1371/journal.ppat.1001242)
Supplement: Table S1 — Accession numbers of redox-related proteins. (0.04 MB DOC) [file ppat.1001242.s002.doc]

**Table S1.** Accession numbers of redox-related proteins.

| **Gene** | **PlasmoDB Access. No.** | **GenBank Access. No.** |
| --- | --- | --- |
| AOP | MAL7P1.159 | AAQ76285 |
| 1-Cys Prx | PF08_0131 | BAA78369 |
| GILP | PFF0230c | CAG25217 |
| GLP1 | PFC0205c | AAK00581 |
| GLP2 | PFF0340c | CAG25239 |
| GLP3 | PF07_0036 | AAC47843 |
| GR | PF14_0192 | CAA63747, HQ399186 |
| tGloII | PFL0285w | AAQ05976 |
| Tlp1 | PF14_0590 | AAQ07982 |
| Tlp2 | PFI1250w | AAQ07983 |
| TPx1 | PF14_0368 | AAF67110 |
| TPx2 | PFL0725w | AAK20024 |
| TPx_Gl_ | PFL0595c | AAN36208 |
| Trx1 | PF14_0545 | AAF34541 |
| Trx2 | MAL13P1.225 | AAQ05974 |
| Trx3 | PFI0790w | AAQ76284 |
| TrxR | PFI1170c | CAA60574, AAQ07981 |
