# Supplementary material for: Compartmentation of Redox Metabolism in Malaria Parasites
Source: PLoS Pathog. 2010 Dec 23;6(12):e1001242. doi: 10.1371/journal.ppat.1001242 (PMC3009606; doi:10.1371/journal.ppat.1001242)
Supplement: Table S2 — Overview of the analyzed redox proteins. (0.18 MB DOC) [file ppat.1001242.s003.doc]

**Table S2.** Overview of the analyzed redox proteins.

| **Gene & References** | **PlasmoDB Access. No.** | **_1_Target Prediction** | | | | **GFP localization** | **Details** |
| --- | --- | --- | --- | --- | --- | --- | --- |
|  |  | **_2_SignalP 3.0**  **SP**  **CS & prob.** | **_3_TargetP 1.1**  **mTP & SP**  **non-plant / plant** | **_4_PlasmoAP**  **SP & aTP** | **_5_PATS** |  |  |
| AOP | MAL7P1.159 | 0.818  20/21 & 0.782 | 0.221 & 0.851  0.157 & 0.915 | ++ & ++  4/4 & 5/5 | 0.836  Yes | Apicoplast | - The first 66 AA fused to GFP |
| 1-Cys Prx | PF08_0131 | 0.039  16/17 & 0.016 | 0.112 & 0.141  0.115 & 0.236 | - & -  0/4 & 2/5 | 0.017  No | Cytosol | - Complete gene fused to GFP |
| GILP | PFF0230c | 0.961  22/23 & 0.532 | 0.119 & 0.881  0.136 & 0.921 | ++ & ++  4/4 & 5/5 | 0.986  Yes | Apicoplast | - The first 66 AA fused to GFP |
| GLP1 | PFC0205c | 0.000  No | 0.692 & 0.023  0.167 & 0.600 | - & ++  1/4 & 5/5 | 0.031  No | Cytosol | - Complete gene fused to GFP |
| GLP2 | PFF0340c | 0.000  No | 0.066 & 0.066  0.166 & 0.099 | - & -  0/4 & 0/5 | 0.025  No | Cytosol | - _6_Complete gene fused to GFP |
| GLP3 | PF07_0036 | 0.018  21/22 & 0.016 | 0.146 & 0.239  0.324 & 0.413 | - & ++  0/4 & 5/5 | 0.0161  No | Mitochon-drion | - Complete gene fused to GFP |
| GR | PF14_0192  (elongated form access. No. HQ399186) | 0.595  20/21 & 0.294 | 0.174 & 0.878  0.068 & 0.985 | ++ & ++  4/4 & 5/5 | 0.009  No | _9_Cytosol & Apicoplast | - AA 44 in N-terminal extension 🡪 K to N mutation in all clones compared to PlasmoDB prediction; PlasmoDB prediction most likely wrong - GR-N71: the first 71 AA - GR-SP: the first 20 AA - GR-TP: AA 21-48 |
| tGloII | PFL0285w | 0.303  17/18 & 0.163 | 0.360 & 0.293  0.323 & 0.943 | 0 & ++  2/4 & 5/5 | 0.260  No | Apicoplast & ER | - _6_Cloned N-terminus comprised the first 96 AA fused to GFP |
| Tlp1 | PF14_0590 | 0.000  No | 0.055 & 0.087  0.037 & 0.189 | - & -  0/4 & 1/5 | 0.023  No | Cytosol | - Complete gene fused to GFP |
| Tlp2 | PFI1250w | 0.000  No | 0.253 & 0.070  0.513 & 0.066 | - & ++  0/4 & 5/5 | 0.004  No | Mitochon-drion | - Complete gene fused to GFP |
| TPx1 | PF14_0368 | 0.000  No | 0.136 & 0.068  0.076 & 0.373 | - & -  0/4 & 2/5 | 0.022  No | Cytosol | - Complete gene fused to GFP |
| TPx2 | PFL0725w | 0.000  No | 0.904 & 0.039  0.862 & 0.037 | - & ++  0/4 & 5/5 | 0.023  No | Mitochon-drion | - Complete gene fused to GFP |
| TPx_Gl_ | PFL0595c | 0.043  21/22 & 0.042 | 0.064 & 0.913  0.043 & 0.895 | ++ & 0  4/4 & 4/5 | 0.769  Yes | Cytosol & Apicoplast | - The first 47 AA fused to GFP |
| Trx1 | PF14_0545 | 0.000  No | 0.043 & 0.204  0.073 & 0.540 | - & -  0/4 & 2/5 | 0.036  No | Cytosol | - Complete gene fused to GFP |
| Trx2 | MAL13P1.225 | 0.788  23/24 & 0.427 | 0.016 & 0.971  0.012 & 0.949 | ++ & -  4/4 & 1/5 | 0.966  Yes | _9_PV & unknown organelle | - The first 66 AA fused to GFP - Complete gene fused to GFP |
| Trx3 | PFI0790w | 0.104  _7_0.856 | 0.026 & 0.966  0.021 & 0.985 | 0 & ++  2/4 & 5/5 | 0.974  Yes | _9_ER | - The first 66 AA fused to GFP - Complete gene fused to GFP - _8_TMHMM v 2.0 predicts 1 N-terminal transmembrane helix (residues 1-6 inside, 7-29 TM helix, 30-179 outside) |
| TrxR | PFI1170c | 0.000  No | 0.312 & 0.043  0.231 & 0.080 | - & ++  0/4 & 5/5 | 0.030  No | _9_Cytosol & Mitochon-drion | - The first 76 AA fused to GFP - _6_Complete gene fused to GFP |

AOP, antioxidant protein; AA, amino acid; aTP, apicoplast targeting peptide; CS, cleave site; ER, endoplasmic reticulum; GILP, glyoxalase-1-like protein; GLP, glutaredoxin-like protein; GR, glutathione reductase; mTP, mitochondrial targeting peptide; PATS, predict apicoplast targeted sequences; PlasmoAP, prediction of apicoplast targeting signals; prob., probability; PV, parasitophorous vacuole; tGloII, targeted glyoxalase II; SignalP, predicts the presence and location of SP cleavage sites in amino acid sequences from different organisms; SP, signal peptide; TargetP, predicts the subcellular location of eukaryotic proteins; Tlp, thioredoxin-like protein; TP, transit peptide; TPx, thioredoxin-dependent peroxidase; Trx, thioredoxin; TrxR, thioredoxin reductase.

1 All predictions made 02-19-2010, except for 1-Cys Prx, Tlp1, Trx1, and TPx1 (predictions were made 09-06-2010)

2 The hidden Markov model (HMM) calculates the probability of whether the submitted sequence contains a signal peptide or not. Furthermore, the cleavage site is assigned by a probability score. For further, detailed information please refer to the citations provided.

3 Final neural network scores on which the final prediction is based (mTP, the sequence contains a mitochondrial targeting peptide; SP, the sequence contains a signal peptide). The scores are not really probabilities, and they do not necessarily add to one. However, the location with the highest score is the most likely according to TargetP, and the relationship between the scores may be an indication of how certain the prediction is. The upper numbers correspond to predictions using the non-plant settings, the lower numbers are predictions using the plant settings. For further, detailed information please refer to the citations provided.

4 For the signal peptide 4 tests are conducted and for the apicoplast targeting peptide 5 tests are conducted (represented by the number x/4 and x/5, respectively). The number on the left indicates the number of positive tests (e.g. 1/4 means that 1 test of 4 for the signal peptide prediction was positive). The final decision is indicated by "++, +, 0 or -", where apicoplast-localization for a given sequence is considered (++ very likely; + likely; 0 undecided; - unlikely). For detailed information please refer to the citation.

5 Neural network output is in [0,1] : values close to 1 are likely apicoplast-targeted; values close to 0 are unlikely apicoplast-targeted.

6 Either a XhoI or a BglII restriction site had to be mutated for cloning purposes. The mutations did not lead to an amino acid exchange.

7 The eukaryotic hidden Markov model (HMM) used by Signal P also reports the probability of a signal anchor. A signal anchor is a domain that is defined by UniProt as follows: “Single-pass transmembrane protein (type II, III, and IV) possessing a membrane-spanning domain which targets the protein to the ER membrane” (http://www.uniprot.org/keywords/735 09-06-2010). This value is provided here because Trx3 is retained in the ER (the prediction was made 09-06-2010 with Signal P). For further, detailed information please refer to the citations provided.

8 TMHMM is a hidden Markov model (HMM) based transmembrane helix (TM) prediction program. The prediction was made 09-06-2010. The program was used because Trx3 is retained in the ER. For further, detailed information please refer to the citations provided.

9 Localization observed for all constructs tested.

**References**

1. Aurrecoechea C, Brestelli J, Brunk BP, Dommer J, Fischer S, et al. (2009) PlasmoDB: a functional genomic database for malaria parasites. Nucleic Acids Res 37: D539-543.

2. Emanuelsson O, Brunak S, von Heijne G, Nielsen H (2007) Locating proteins in the cell using TargetP, SignalP and related tools. Nat Protoc 2: 953-971.

3. Bendtsen JD, Nielsen H, von Heijne G, Brunak S (2004) Improved prediction of signal peptides: SignalP 3.0. J Mol Biol 340: 783-795.

4. Nielsen H, Engelbrecht J, Brunak S, von Heijne G (1997) Identification of prokaryotic and eukaryotic signal peptides and prediction of their cleavage sites. Protein Eng 10: 1-6.

5. Emanuelsson O, Nielsen H, Brunak S, von Heijne G (2000) Predicting subcellular localization of proteins based on their N-terminal amino acid sequence. J Mol Biol 300: 1005-1016.

6. Foth BJ, Ralph SA, Tonkin CJ, Struck NS, Fraunholz M, et al. (2003) Dissecting apicoplast targeting in the malaria parasite Plasmodium falciparum. Science 299: 705-708.

7. Zuegge J, Ralph S, Schmuker M, McFadden GI, Schneider G (2001) Deciphering apicoplast targeting signals--feature extraction from nuclear-encoded precursors of Plasmodium falciparum apicoplast proteins. Gene 280: 19-26.

8. Nickel C, Rahlfs S, Deponte M, Koncarevic S, Becker K (2006) Thioredoxin networks in the malarial parasite Plasmodium falciparum. Antioxid Redox Signal 8: 1227-1239.

9. Sarma GN, Nickel C, Rahlfs S, Fischer M, Becker K, et al. (2005) Crystal structure of a novel Plasmodium falciparum 1-Cys peroxiredoxin. J Mol Biol 346: 1021-1034.

10. Kawazu S, Tsuji N, Hatabu T, Kawai S, Matsumoto Y, et al. (2000) Molecular cloning and characterization of a peroxiredoxin from the human malaria parasite Plasmodium falciparum. Mol Biochem Parasitol 109: 165-169.

11. Akoachere M, Iozef R, Rahlfs S, Deponte M, Mannervik B, et al. (2005) Characterization of the glyoxalases of the malarial parasite Plasmodium falciparum and comparison with their human counterparts. Biol Chem 386: 41-52.

12. Rahlfs S, Fischer M, Becker K (2001) Plasmodium falciparum possesses a classical glutaredoxin and a second, glutaredoxin-like protein with a PICOT homology domain. J Biol Chem 276: 37133-37140.

13. Deponte M, Becker K, Rahlfs S (2005) Plasmodium falciparum glutaredoxin-like proteins. Biol Chem 386: 33-40.

14. Farber PM, Arscott LD, Williams CH, Jr., Becker K, Schirmer RH (1998) Recombinant Plasmodium falciparum glutathione reductase is inhibited by the antimalarial dye methylene blue. FEBS Lett 422: 311-314.

15. Urscher M, Przyborski JM, Imoto M, Deponte M (2010) Distinct Subcellular Localization in the Cytosol and Apicoplast, Unexpected Dimerization, and Inhibition of Plasmodium falciparum Glyoxalases. Mol Microbiol 76: 92-103.

16. Muller S (2004) Redox and antioxidant systems of the malaria parasite Plasmodium falciparum. Mol Microbiol 53: 1291-1305.

17. Rahlfs S, Nickel C, Deponte M, Schirmer RH, Becker K (2003) Plasmodium falciparum thioredoxins and glutaredoxins as central players in redox metabolism. Redox Rep 8: 246-250.

18. Boucher IW, McMillan PJ, Gabrielsen M, Akerman SE, Brannigan JA, et al. (2006) Structural and biochemical characterization of a mitochondrial peroxiredoxin from Plasmodium falciparum. Mol Microbiol 61: 948-959.

19. Sztajer H, Gamain B, Aumann KD, Slomianny C, Becker K, et al. (2001) The putative glutathione peroxidase gene of Plasmodium falciparum codes for a thioredoxin peroxidase. J Biol Chem 276: 7397-7403.

20. de Koning-Ward TF, Gilson PR, Boddey JA, Rug M, Smith BJ, et al. (2009) A newly discovered protein export machine in malaria parasites. Nature 459: 945-949.

21. Sonnhammer EL, von Heijne G, Krogh A (1998) A hidden Markov model for predicting transmembrane helices in protein sequences. Proc Int Conf Intell Syst Mol Biol 6: 175-182.

22. Krogh A, Larsson B, von Heijne G, Sonnhammer ELL (2001) Predicting transmembrane protein topology with a hidden Markov model: Application to complete genomes. Journal of Molecular Biology 305: 567-580.
