# Supplementary material for: Compartmentation of Redox Metabolism in Malaria Parasites
Source: PLoS Pathog. 2010 Dec 23;6(12):e1001242. doi: 10.1371/journal.ppat.1001242 (PMC3009606; doi:10.1371/journal.ppat.1001242)
Supplement: Table S3 — Oligonucleotide primers. (0.10 MB DOC) [file ppat.1001242.s004.doc]

**Table S3.** Oligonucleotide primers.

| **Gene** | **PlasmoDB**  **Access. No.** | **Construct** | **Orientation** | **Sequence** |
| --- | --- | --- | --- | --- |
| **AOP** | **MAL7P1.159** | AOP | sense | TTAAGATCTATGAGAATGAGAAGAACAATAC |
|  |  | N-term AOP | antisense | AACCTAGGacctgctgcAGGAATAAGATCATTTTCTTTTA |
| **1-Cys**  **Prx** | **PF14_0590** | 1-Cys Prx | sense | TTAAGATCTATGGCTTACCATTTAGGAGCTAC |
|  |  | 1-Cys Prx complete | antisense | AACCCTAGGACCTGCTGCCATTTGAACAAATCTTAAATATGC |
| **GILP** | **PFF0230c** | GILP | sense | TTAAGATCTATGAAACTTTTTGTAGTCGTTAT |
|  |  | N-term GILP | antisense | AACCCTAGGacctgctgcTGATTTGTCTAAATCATGGACC |
| **GLP1** | **PFC0205c** | GLP1 | sense | TTAAGATCTATGATAATGAAAAACAAATATGGAG |
|  |  | GLP1 complete | antisense | AACCCTAGGacctgctgcTTTTATAATTTTTTCCAACTC |
| **GLP2** | **PFF0340c** | GLP2 | sense | TTAAGATCTATGGATTTTATTAAGGTTGAG |
|  |  | GLP2 complete | antisense | AACCCTAGGacctgctgcTTCTTCGAAACAGTCATCTGG |
|  |  | GLP2 XhoI Mut | sense | gatatcaattgttttacctaaactccagtacatctaaagaatatggttctc |
|  |  | GLP2 XhoI Mut | antisense | gagaaccatattctttagatgtactggagtttaggtaaaacaattgatatc |
| **GLP3** | **PF07_0036** | GLP3 | sense | TTAAGATCTATGAACAAATACATAAGAGCGC |
|  |  | GLP3 complete | antisense | AACCCTAGGacctgctgcTATAATATCTTTAATTTTATTTTGG |
| **GR** | **PF14_0192** | GR | sense | CATGGATCCATGTACAAACATAGATACTTTC |
|  |  | N-term GR | antisense | CATCCTAGGacctgctgcGTTATGCCTTGCTGCCC |
|  |  | Signal peptide GR | sense | TTAAGATCTATGATGTACAAACATAGATA |
|  |  | Signal peptide GR | antisense | AACCCTAGGacctgctgcGGTTGACACGAGAAAAA |
|  |  | Target peptide GR | sense | TTAAGATCTATGAAAATAATAAGAAGTTTTACT |
|  |  | Target peptide GR | antisense | AACCCTAGGacctgctgcAACCATATTTGCGTTTTTTTTA |
|  |  | N-term GR M47A Mut | sense | CAGTATACTTTAAAAAAAACGCAAATGCGGTTTACGATTTAATTG |
|  |  | N-term GR M47A Mut | antisense | CAATTAAATCGTAAACCGCATTTGCGTTTTTTTTAAAGTATACTG |
| **tGloII** | **PFL0285w** | tGloII | sense | TTAAGATCTATGAGATTTCTTAAAACATTATTT |
|  |  | N-term tGloII | antisense | AACCCTAGGacctgctgcCTTAGATATATCATTTATTATATTATA |
|  |  | tGloII BglII Mut | sense | GAAAATTCTTTTATAAGAAAAGATTTACATAC |
|  |  | tGloII BglII Mut | antisense | GTATGTAAATCTTTTCTTATAAAAGAATTTTC |
| **Tlp1** | **PF14_0590** | Tlp1 | sense | TTAAGATCTATGAAACGAACTGACGATAAAATTTAC |
|  |  | Tlp1 complete | antisense | AACCCTAGGACCTGCTGCATTTATTTTCTTATTCATCAAATGC |
| **Tlp2** | **PFI1250w** | Tlp2 | sense | TTAAGATCTATGTTTTTTCTTCAAAATTTAAAAAA |
|  |  | Tlp2 complete | antisense | AACCCTAGGacctgctgcATCACTCTGATGTTTTTC |
| **TPx1** | **PF14_0368** | TPx1 | sense | TTAAGATCTATGGCATCATATGTAGGAAGAGAAG |
|  |  | TPx1 complete | antisense | AACCCTAGGACCTGCTGCCAACTTTGATAAATATTCACTAAC |
| **TPx2** | **PFL0725w** | TPx2 | sense | TTAAGATCTATGTTTTTAAAAAAACTGTGCAGGAGC |
|  |  | TPx2 complete | antisense | AACCCTAGGacctgctgcTACATTTTTATTTGCATTATTCATA |
| **TPx_Gl_** | **PFL0595c** | TPx_Gl_ | sense | TTAAGATCTATGTTCTTCTCAATGTTTATTAAATTC |
|  |  | N-term TPx_Gl_ | antisense | AACCTAGGacctgctgcATAATCATAAATGGATGATAATAATTC |
| **Trx1** | **PF14_0545** | Trx1 | sense | TTAAGATCTATGGTAAAAATTGTAACTAGTCAAGC |
|  |  | Trx1 complete | antisense | AACCCTAGGACCTGCTGCAGCTGCGTATTTTTCGATAAG |
| **Trx2** | **MAL13P1.225** | Trx2 | sense | TTAAGATCTATGAAGAAGTATATATTTTTCTTTCTC |
|  |  | N-term Trx2 | antisense | AACCCTAGGacctgctgcAACACCGTTGATAATATTTGATCC |
|  |  | Trx2 complete | antisense | AACCCTAGGacctgctgcTAAATGTTTTTTAATTAATGCTATC |
| **Trx3** | **PFI0790w** | Trx3 | sense | TTAAGATCTATGGCTCTTATTTGCATCGG |
|  |  | N-term Trx3 | antisense | AACCCTAGGacctgctgcACTTTGTTCAAGCTGCTTAT |
|  |  | Trx3 complete | antisense | AACCCTAGGacctgctgcTTTTGCTTTTTCTAAACAG |
| **TrxR** | **PFI1170c** | TrxR | sense | CATGGATCCATGAACAATGTAATTTCTTTCATTGG |
|  |  | N-term TrxR | antisense | CATCCTAGGacctgctgcGTTTTTAATGGAAAAGTTTAATTTGG |
|  |  | TrxR complete | antisense | CATCCTAGGacctgctgcTCCACATTTTCCACCCCCACATCC |
|  |  | TrxR XhoI Mut | sense | CACATGGTGCACGAGTTTTATTGTTTG |
|  |  | TrxR XhoI Mut | antisense | CAAACAATAAAACTCGTGCACCATGTG |
