# Supplementary figures and images for: Compartmentation of Redox Metabolism in Malaria Parasites
Source: PLoS Pathog. 2010 Dec 23;6(12):e1001242. doi: 10.1371/journal.ppat.1001242 (PMC3009606; doi:10.1371/journal.ppat.1001242)

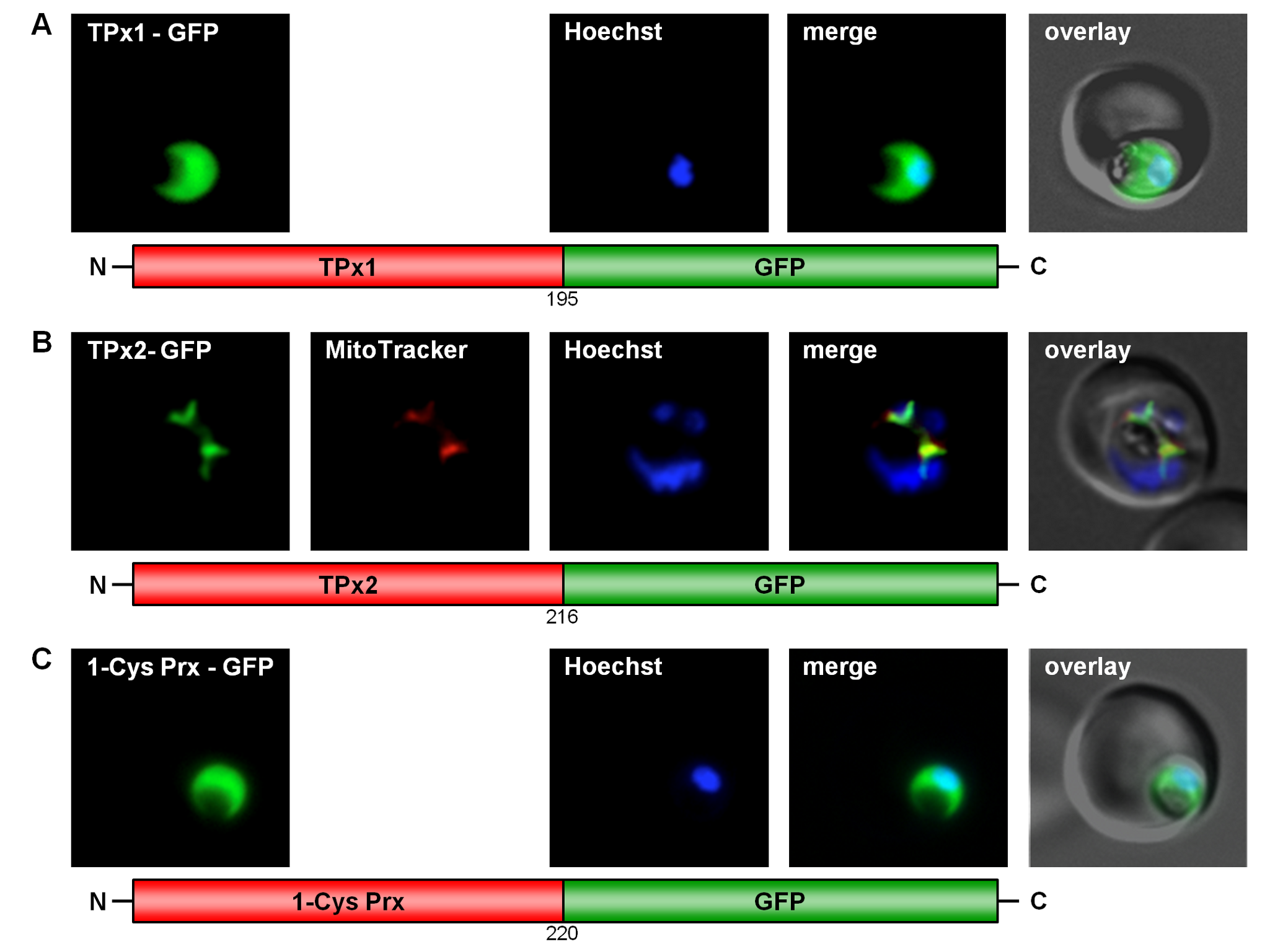

Supplement: Figure S1 — GFP targeting by various P. falciparum peroxiredoxins. (A) Cytosolic localization of TPx1. (B) Mitochondrial targeting of TPx2. (C) Cytosolic localization of 1-Cys Prx. Live cell imaging of erythrocytes infected with transgenic parasites for solely cytosolic GFP signals. Colocalization of GFP with the mitochondrial stain MitoTrackerOrange in fixed cells. (0.63 MB TIF) [file ppat.1001242.s005.tif]

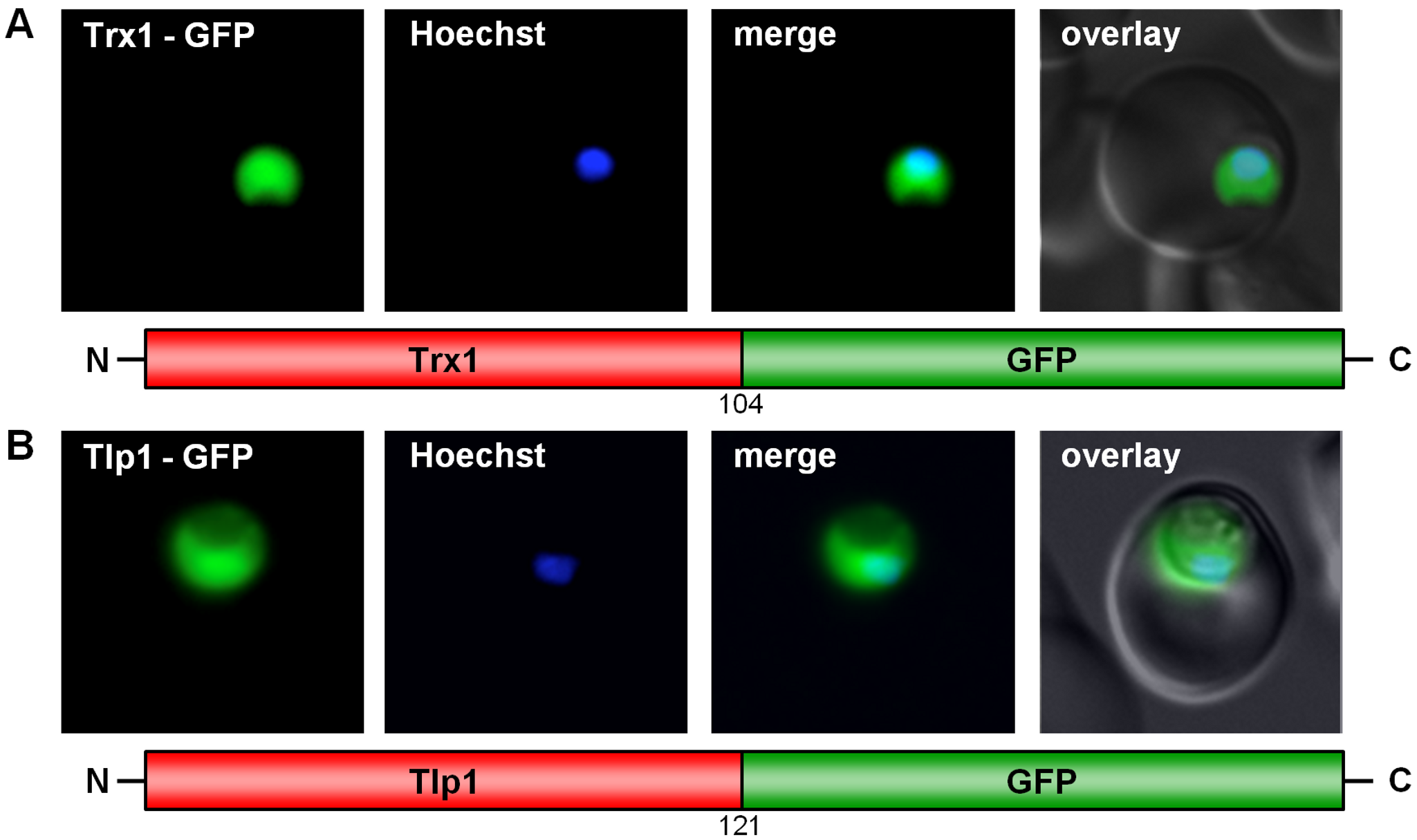

Supplement: Figure S2 — GFP targeting by P. falciparum thioredoxin 1 and thioredoxin-like protein 1. (A) Cytosolic localization of Trx1. (B) Cytosolic localization of Tlp1. Live cell imaging of erythrocytes infected with transgenic parasites for solely cytosolic GFP signals. (0.48 MB TIF) [file ppat.1001242.s006.tif]

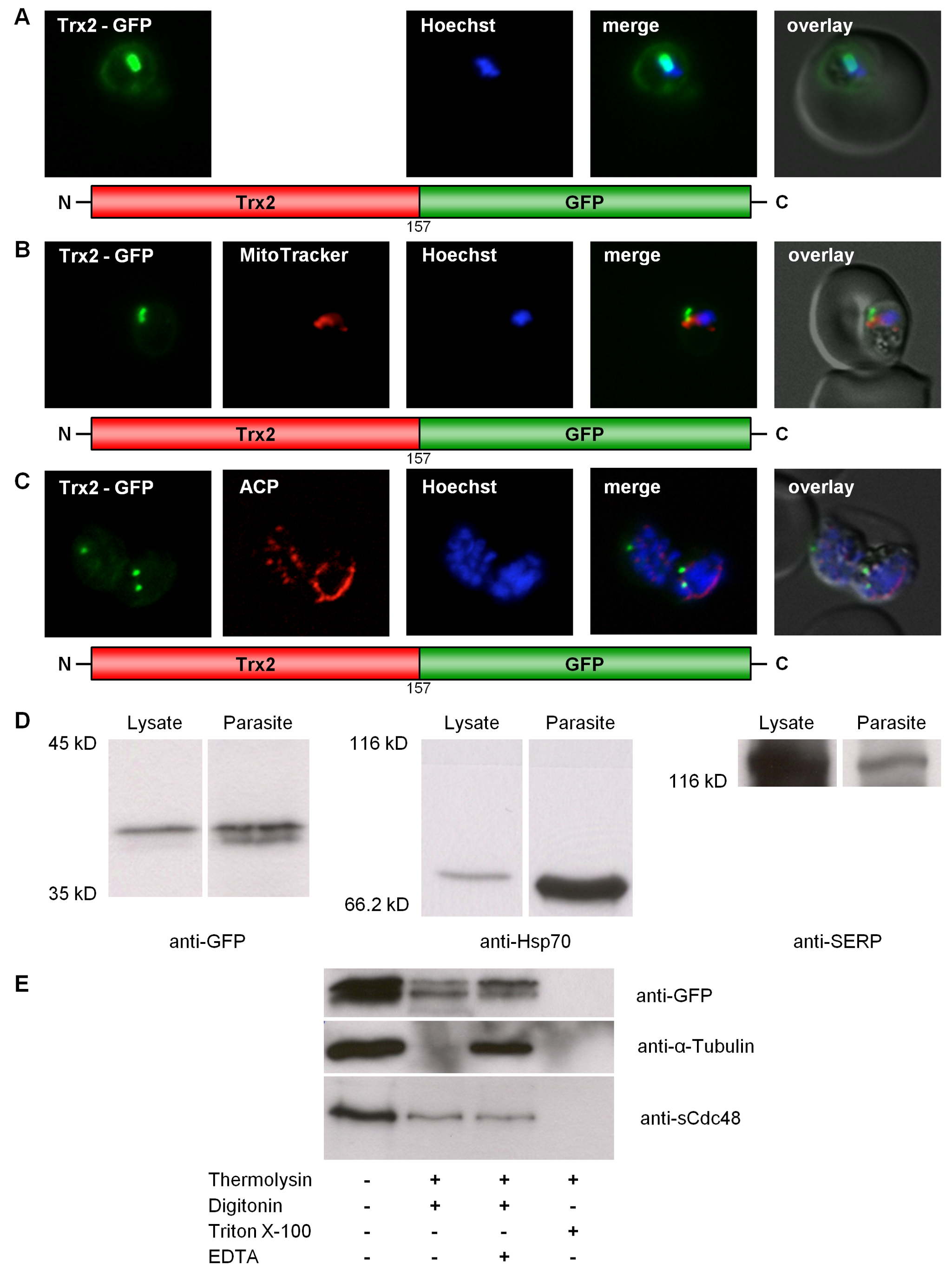

Supplement: Figure S3 — GFP targeting by P. falciparum thioredoxin 2. (A)–(C) Targeting of Trx2 to the parasitophorous vacuole and to a not yet characterized, non-dividing organelle within the parasite. (D) Western blot analysis of parasites stably expressing Trx2-GFP using anti-GFP, anti-Hsp70, and anti-SERP antibodies confirms dual localization of Trx2 to the parasitophorous vacuole and the cellular part of parasite. Lysate, erythrocyte cytosol plus the soluble contents of the parasitophorous vacuole; Parasite, cellular contents of the parasite. (E) Thermolysin protection assays on parasites stably expressing Trx2-GFP confirm organellar localization of Trx2. Parasites permeabilized using the detergents digitonin (plasma membrane) and Triton X-100 (plasma membrane and organellar membranes) were treated with the protease thermolysin. Tubulin is the cytosolic control that is not protected from thermolysin after digitonin permeabilization; sCdc48 is the apicoplast-targeted control protein that is protected from thermolysin after dititonin permeabilization but not after Triton X-100 permeabilization. Degradation could be inhibited with the addition of EDTA, an inhibitor of thermolysin, suggesting that the loss of protein we observed was specifically due to thermolysin degradation. Colocalization of GFP with the mitochondrial stain MitoTrackerOrange in fixed cells. Colocalization of GFP and the apicoplast marker ACP in fixed, immunodecorated cells. (1.72 MB TIF) [file ppat.1001242.s007.tif]

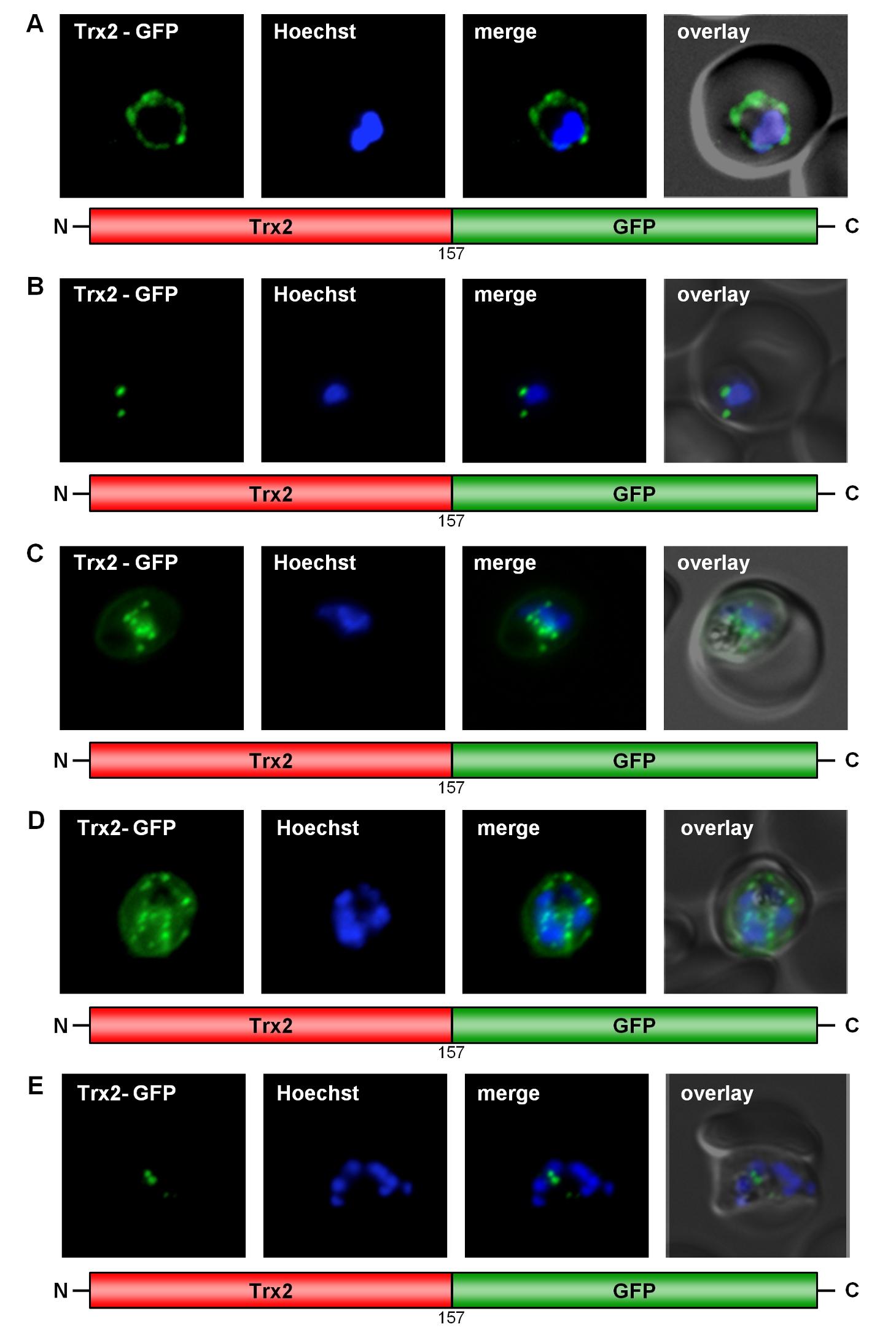

Supplement: Figure S4 — GFP targeting by thioredoxin 2 in various stages of P. falciparum. (A) Early ring stage parasite showing Trx2-GFP targeting to the PV. (B) Early trophozoite stage parasite showing two fluorescent Trx2-GFP points lying directly under the parasite plasma membrane. (C) Late trophozoite/early schizont stage parasite showing numerous fluorescent Trx2-GFP points. (D) Schizont stage parasite showing numerous fluorescent Trx2-GFP points. (E) Schizont stage parasite showing a small number of observable fluorescent Trx2-GFP structures. Live cell imaging of erythrocytes infected with transgenic parasites. (0.69 MB TIF) [file ppat.1001242.s008.tif]

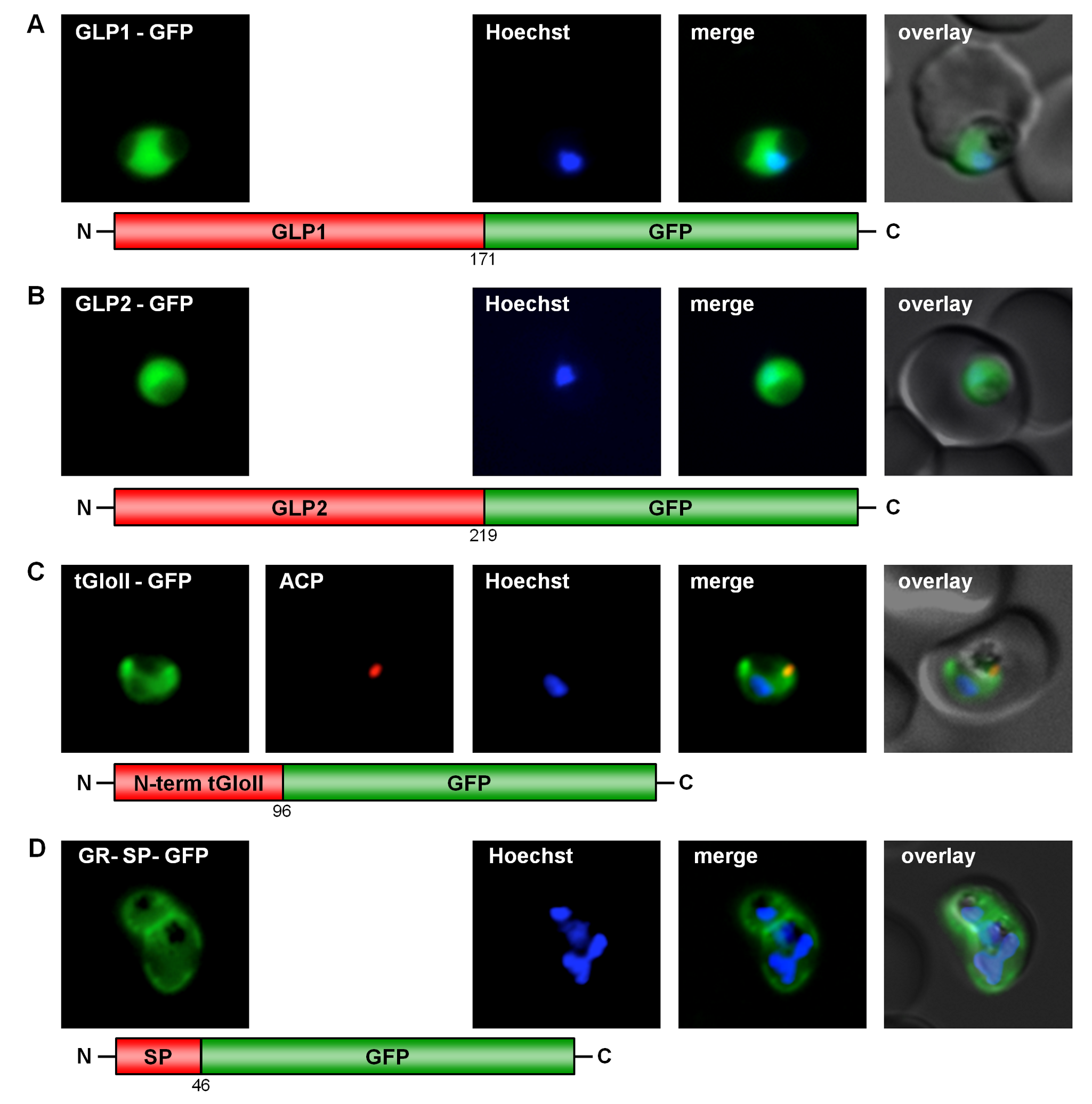

Supplement: Figure S5 — GFP targeting by various P. falciparum redox proteins of the glutathione system. (A) Cytosolic localization of GLP1. (B) Cytosolic localization of GLP2. (C) Dual localization (cytosol and apicoplast) of the tGloII N-terminus. (D) PV-localization of the signal peptide of GR (the parasites showed a fast bleaching fluorescence, leading to a high background signal). Live cell imaging of erythrocytes infected with transgenic parasites for solely cytosolic GFP signals. Colocalization of GFP and the apicoplast marker ACP in fixed, immunodecorated cells. (0.89 MB TIF) [file ppat.1001242.s009.tif]
